# Cyst Reduction in a Polycystic Kidney Disease *Drosophila* Model Using Smac Mimics

**DOI:** 10.3390/biomedicines7040082

**Published:** 2019-10-18

**Authors:** Cassandra Millet-Boureima, Ramesh Chingle, William D. Lubell, Chiara Gamberi

**Affiliations:** 1 Biology Department, Concordia University, Montreal, QC H4B 1R6, Canada; cassandra.millet@mail.concordia.ca; 2 Département de Chimie, Université de Montréal, Montreal, QC H3T 1J4, Canada; ramesh.chingle@nih.gov (R.C.); lubell@chimie.umontreal.ca (W.D.L.)

**Keywords:** renal cystogenesis, *Drosophila*, disease models, Smac mimicry, polycystic kidney disease, azapeptide

## Abstract

Autosomal dominant polycystic kidney disease (ADPKD) is an inherited malady affecting 12.5 million people worldwide. Therapeutic options to treat PKD are limited, due in part to lack of precise knowledge of underlying pathological mechanisms. Mimics of the second mitochondria-derived activator of caspases (Smac) have exhibited activity as antineoplastic agents and reported recently to ameliorate cysts in a murine ADPKD model, possibly by differentially targeting cystic cells and sparing the surrounding tissue. A first-in-kind *Drosophila* PKD model has now been employed to probe further the activity of novel Smac mimics. Substantial reduction of cystic defects was observed in the Malpighian (renal) tubules of treated flies, underscoring mechanistic conservation of the cystic pathways and potential for efficient testing of drug prototypes in this PKD model. Moreover, the observed differential rescue of the anterior and posterior tubules overall, and within their physiologically diverse intermediate and terminal regions implied a nuanced response in distinct tubular regions contingent upon the structure of the Smac mimic. Knowledge gained from studying Smac mimics reveals the capacity for the *Drosophila* model to precisely probe PKD pharmacology highlighting the value for such critical evaluation of factors implicated in renal function and pathology.

## 1. Introduction

Autosomal dominant polycystic kidney disease (ADPKD) induces the formation of cysts along the entire renal tubule, predominantly at the terminal region and the collector tubule, as well as extra-renal manifestations [1]. Inherited through a monogenic pattern with the most frequent mutations affecting the *PKD1* or *PKD2* gene, ADPKD displays high heterogeneity in both phenotype and speed of progression [1]. Abnormal proliferation of the epithelial tubular cells during development gives rise to cysts prenatally. Cysts become more numerous with age, gradually enlarging and filling with fluid [1]. Animal models of PKD have been invaluable for defining disease progression and identifying key molecular alterations; however, the precise mechanisms underlying disease pathology remain to be elucidated at the molecular level [2]. The most recent addition to the arsenal of PKD animal models has been the fruit fly, *Drosophila melanogaster*. Mutants for the *Bicaudal C* (*BicC*) gene (hereby *BicC* flies) were found to recapitulate key molecular features of PKD, including *myc* over-expression and mechanistic target of rapamycin (mTOR) pathway activation [3]. Consistent with ADPKD, the *BicC* flies formed cysts along the entire length of the tubule, with higher frequency at the intermediate, terminal and collector tubule regions [3]. Moreover, analogous to vertebrate PKD [4,5,6,7,8], pharmacological treatment of *BicC* flies with rapamycin transiently reduced cysts [3]. Consistent with the relevance of the *BicC* phenotype in PKD, *BICC1* mRNA and Bicc1 protein orthologues were respectively found to decrease in kidneys from *PKD1* patients and *Pkd1^-/-^* mice [3]. Thus, *PKD1* dysfunction is associated with decreased *BicC* function.

An excellent model of human renal function, the fly has high genetic conservation and a streamlined anatomy (reviewed in [9]). In contrast to the human kidney, which contains one million tubular filtering units called nephrons, *Drosophila* harbors two pairs of Malpighian tubules (MTs), which are functionally equivalent to the tubular portion of the nephron. Suitable to the open circulatory system of the fly, the renal system does not have glomeruli and possesses nephrocytes, which exhibit re-adsorptive function analogous to the human glomerular podocytes [9]. Originating from the interface between the mid- and hind-gut, the MTs are asymmetrical with longer tubules anteriorly and shorter ones posteriorly. Reminiscent of the different nephron types in the human kidney, the anterior and posterior MTs have distinct transcriptomes [10]. Like the human renal tubules, MTs can be divided into distinct regions, which in the fly are called proximal, intermediate, and terminal. The proximal region excretes fluid into the tubules, the intermediate region secretes potassium chloride and water, and the terminal region is responsible for sodium and, possibly, water reabsorption [9]. One key advantage of the fly renal system is that the MTs are anatomically distinct, float freely in the fly body cavity, and can be cleanly micro-dissected and examined in their entirety.

Antineoplastic mimics of the second mitochondria-derived activator of caspases (Smac, also called the direct inhibitor of apoptosis-binding protein with low pI, DIABLO) have been used to sensitize cancer cells to apoptosis by targeting inhibitors of apoptosis proteins (IAPs, [11,12]). Highly conserved, IAP proteins play a key role in balancing cell survival and cell death through multiple intersecting cellular pathways. Implicated in innate immunity [13,14], IAPs are often upregulated in cancer [15]. First discovered in insect baculovirus as inhibitors of cellular apoptosis which enable viral replication, IAPs were found in both vertebrates and invertebrates [16] (reviewed in [11]). The best-known mammalian IAPs are X-linked IAP (XIAP), cellular IAP1 (cIAP1) and cIAP2 [11]. The *Drosophila* genome encodes four IAPs, Diap1, Diap2, dBruce and Deterin [17,18,19]. Multiple proteins have been shown to antagonize IAPs. First discovered in *Drosophila*, three proteins, Reaper [20], Head involution defective (Hid, [21]) and Grim [22], can induce apoptosis when transfected into mammalian cells, which demonstrated functional conservation of the apoptotic cellular machinery [23]. Additional fly IAP antagonists are Jafrac [24], Sickle [25,26,27] and HtrA2 [28,29,30]. The search for the mammalian IAP antagonists yielded Smac/DIABLO [31,32], Omi/HtrA2 [33], apoptosis-related protein in the TGFβ signaling pathway (ARTS, [34]) and XIAP-associated factor 1 (XAF1, [35]). IAP antagonists function through an RHG motif, named after the prototypical Reaper, Hid and Grim fly proteins. In the case of the well-studied human Smac/DIABLO and fly Hid IAP antagonists, pro-apoptotic stimuli induce proteolytic cleavage exposing the RHG motif at the *N*-terminus enabling interaction with IAP proteins [36,37] and triggering their ubiquitination and degradation. In the appropriate cellular context, IAP degradation can eventually lead to cell death either by way of caspase activation or via tumor necrosis factor (TNF) signaling [11,38,39]. The discovery that the Smac/DIABLO *N*-terminal peptide H-Ala-Val-Pro-Ile-NH_2_ can recapitulate the pro-apoptotic function of the entire protein has inspired the development of various Smac mimics, which enable sensitization of neoplastic cells to apoptosis [12].

Based on the premise that ADPKD is considered in part a neoplastic condition [40,41], and that various TNF complex components were upregulated in *Pkd1^-/-^* mouse embryonic kidney (MEK) cells, the Smac mimic and birinapant analog GT13072 was used to induce TNF-α-dependent cell death in both cultured cells and a rat model of PKD [42]. The pro-apoptotic properties of GT13072 were specific for the TNF-positive renal epithelial cystic cells and spared surrounding non-cystic cells, offering therapeutic potential to ablate renal cystic cells to delay cystogenesis [42].

**Figure 1:**
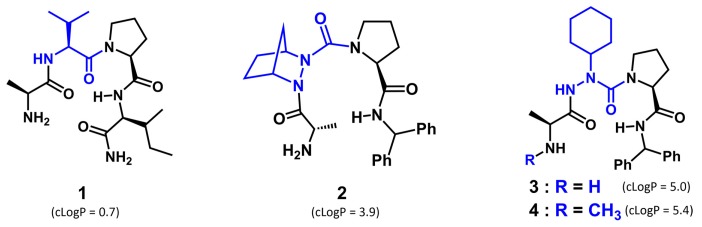
Second mitochondria-derived activator of caspases (Smac) analogs: peptide **1** and mimics **2**–**4** (Val, aza-residue and modified ***N***-terminal in blue). Logarithm of the partition coefficient (clogP) values were calculated as described below.

The TNF pathway is highly conserved in the fly [43,44,45,46,47,48]. The capacity for Smac mimics to reduce MT cystogenesis has now been tested in the *BicC* cystic flies. The prototypical H-Ala-Val-Pro-Ile-NH_2_ (peptide **1**, Figure 1) and three Smac mimics **2**–**4** were administered to the *BicC* cystic flies to reduce cyst formation.

## 2. Experimental Section

### 2.1. Fly Lines and Genetics

Fly breeding and care were previously described in detail [3]. In brief, flies were grown on cornmeal agar (Jazzmix, Fisher Scientific Canada, Ottawa, ON) at 25 °C and aged as indicated. *Oregon^R^* (*Ore^R^*) wild-type flies were maintained as in [3]. *BicC* mutants were generated by crossing *Df(2L)RA5*/*CyO* (*BicC-*encompassing deletion obtained from Bloomington *Drosophila* Stock Center) virgin females with one of the two hypomorphic *BicC* mutations, *BicC^YC33^/CyO* and *BicC^IIF34^/CyO* and selecting straight-winged progeny (*Df(2L)RA5*/*BicC^YC33^, BicC^Δ/YC33^* and *Df(2L)RA5*/*BicC^IIF34^, BicC^Δ/IIF34^*). The two *BicC* allelic combinations produced truncated proteins and sterile *BicC* flies [3]. Eclosed adult flies were collected every two days to yield 0–2-day old populations and aged as described.

### 2.2. In Vivo and Ex Vivo Assays

#### 2.2.1. Cystic Index

For the cyst analysis, 0–2-days old flies were aged seven days (i.e., 7–9 days old) and were fed every three days with 2 mL of cornmeal food and equal volumes (50 µL) of either vehicle (water) or an aqueous solution containing peptide **1** or Smac mimic **2**–**4**. Each compound was used at 20 µM and was administered for 20 days (i.e., until 27–29 days old). Malpighian tubules were micro-dissected from 25–50 female flies in phosphate buffered saline (PBS) and the number of cysts was scored separately in anterior and posterior tubules and assigned to each tubular region (i.e., proximal, intermediate and terminal [3,9]), as follows. At one extremity, the proximal region consists of about 15% of the tubule length, tends to have an opaque whitish content (posterior tubule), is thinner than adjacent regions (especially in the anterior tubule) and often exhibits a slight constriction terminally. The neighboring intermediate region has darker contents and consists of about 55% of the tubule length. The terminal region is often translucent, directly connected to the collecting tubule, and consists of the remaining 30% of the tubule length. Cysts were considered tubular deformations creating uni- or bi-laterally protuberant pouches. Extra-branches (as in [3]) were counted as cysts. Values were plotted using the Prism 8.0 software (Graphpad Software, San Diego, CA, USA) as nested distributions. Statistical analyses were performed as unpaired t tests with Welch’s correction (the populations may not have equal standard deviations). The raw data counts are listed in Supplementary Table S1.

#### 2.2.2. Microscopy

Malpighian tubules from appropriately aged and treated flies were micro-dissected in 1× PBS, equilibrated into a 3:1 1× PBS:glycerol solution and photographed on a Leica MZ FLIII Fluorescence Stereomicroscope with Leica MZ series 10×/21B Widefield adjustable eyepieces equipped with a Canon DS126201 EOS 5D MARK II camera, using visible light. Canon raw files (CR2) were converted into TIF format using the Adobe Lightroom 3.2 software (Adobe Systems, San Jose, CA, USA).

### 2.3. General Synthetic Methods

Chemicals were used as received from commercial sources without further purification unless stated otherwise. All glassware was stored in the oven or flame-dried and let cool under an inert atmosphere prior to use. Anhydrous solvents (DCM, and DMF) were obtained by passage through solvent filtration systems (Glass-Contour, Irvine, CA, USA). Silica gel chromatography was performed using 230–400 mesh silica gel (Silicycle), and TLC was on glass-backed silica plates visualizing the developed chromatogram by UV absorbance or staining with ceric ammonium molybdate or potassium permanganate solutions. Nuclear magnetic resonance spectra (^1^H and ^13^C) were recorded on a Bruker AV 500 spectrometer and referenced to residual solvent in CD_3_OD (3.31 ppm, 49.0 ppm). Coupling constant *J* values and chemical shifts were measured in Hertz (Hz) and parts per million (ppm). Infrared spectra were recorded in the neat on a Perkin Elmer Spectrum One FTIR instrument and are reported in reciprocal centimeter (cm^–1^). Liquid chromatography−mass spectrometry (LC−MS) was performed on an Agilent Technologies 1200 series instrument in positive electrospray ionization (ESI)-time-of-flight (TOF) mode at the Université de Montréal Mass Spectrometry Facility. Sodium and proton adducts ([M + Na]^+^ and [M + H]^+^) were used for empirical formula confirmation. The peptide H-Ala-Val-Pro-Ile-NH_2_ (**1**), Smac mimics **2** and **3**, and aza-cyclohexylglycinyl-l-proline benzhydrylamide (**8**), all were synthesized according to published methods [49]. *N*-Boc-*N*-Methyl-l-alanine and diisopropyl ethyl amine (DIEA) were purchased from Aldrich or Alfa Aesar and used without further purification. Benzotriazol-1-yl-oxytripyrrolidino-phosphoniumhexafluoro-phosphate (PyBop) was purchased from GL Biochem™, recrystallized prior to use from dry CH_2_Cl_2_/Et_2_O (melting point, 156 °C), and stored in the dark.

*N*-Methyl alaninyl-aza-cyclohexylglycinyl-l-proline benzhydrylamide (**4**). A solution of aza-cyclohexylglycinyl-l-proline benzhydrylamide (**8**, 1 eq., 65 mg, 0.155 mmol, prepared according to [49]) and DIEA (2 eq., 40 mg, 53 µL, 0.309 mmol) was added to a solution of *N*-(*tert*-butoxycarbonyl)-*N*-methyl-l-alanine (1.2 eq., 38 mg, 0.186 mmol) and PyBOP (1.5 eq., 121 mg, 0.233 mmol) in DMF (3 mL), and the mixture was stirred overnight. The volatiles were removed under vacuum. The residue was dissolved in EtOAc (10 mL), washed with 5 mL of saturated aqueous NaHCO_3_ and brine (10 mL), dried over Na_2_SO_4_, filtered, and evaporated. Without further purification, the residue was dissolved in a 25% solution of trifluoroacetic acid in dichloromethane (2 mL) and stirred for 2 h. The volatiles were removed under vacuum. The residue was dissolved in CH_2_Cl_2_ and the solution was evaporated. The residue was suspended in 2 mL of 1N HCl, stirred for 30 min, and freeze-dried to give the hydrochloride salt as off white solid, which was purified by RP-HPLC on a reverse-phase Gemini^®^ C18 column (Phenomenex^®^ Inc., pore size: 110 Å, particle size: 5 μm, 250 × 21.2 mm) using a binary solvent system consisting of a gradient of 5–60% MeOH [0.1% formic acid (FA)] in water (0.1% FA) with a flow rate of 10.0 mL/min and UV detection at 214 nm. The desired fractions were combined and freeze-dried to white fluffy powder: azapeptide **4** (5.2 mg, 0.01 mmol, 7%): mp 93–94 °C; ^1^H NMR (500 MHz, CD_3_OD) δ 8.40 (s, 1H), 7.45–7.20 (m, 10H), 6.24 (s, 1H), 4.56–4.42 (m, 1H), 4.05–3.88 (m, 1H), 3.69–3.42 (m, 2H), 2.59–2.43 (m, 3H), 2.43–2.31 (m, 1H), 2.26–2.14 (m, 1H), 2.14–2.00 (m, 1H), 2.00–1.91 (m, 1H), 1.91–1.62 (m, 6H), 1.53–1.36 (m, 5H), 1.35–1.03 (m, 5H); ^13^C NMR (125 MHz, MeOD) δ 174.1, 171.2, 161.4, 143.2, 139.1, 129.5 (2C), 129.4 (2C), 129.3 (2C), 128.8 (2C), 128.4, 128.2, 63.6, 57.9, 57.4, 49.6, 31.5, 31.3, 30.8, 26.9, 26.72 (2C), 26.69 (2C), 26.5, 17.5; IR (neat) vmax/cm^−1^ 2929, 1638, 1532, 1495, 1449, 1406, 1344, 1323, 1095; HRMS m/z calculated for C_29_H_40_N_5_O_3_ [M+H]^+^ 506.3126; found 506.3139. (Supplementary File S1). The logarithm of the partition coefficient (clogP) values were calculated using Chemdraw 17.0 (Perkin Elmer, 2017, Waltham, MA, USA).

## 3. Results

### 3.1. Chemistry

**Scheme 1:**
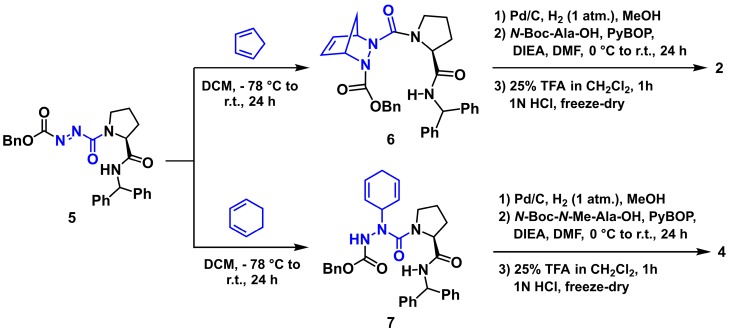
Synthesis of Smac mimics **2** and **4** (dienes, azo- and aza-residues in blue).

Smac activity has been correlated to binding to IAP proteins and mimicked by its *N*-terminal four residue amide sequence (H-Ala-Val-Pro-Ile-NH_2_, **1**, Figure 1) [50,51,52]. The purported turn conformation adopted about the central Val-Pro dipeptide in this sequence has led to the synthesis of various constrained analogs, exhibiting enhanced potency [12,53]. Noting the similar conformational preferences of indolizidinone amino acid and aza-amino acyl proline turn mimics [54,55,56,57], and the relative ease of synthesis of the latter, a series of aza-analogs were synthesized and certain were shown to induce cell death by a caspase-9 mediated apoptotic pathway in cancer cell cultures [49,57]. Notably, aza-methanopipecolate and aza-cyclohexylglycine analogs **2** and **3** were synthesized using pericyclic chemistry on the diazo dicarbonyl moiety of an azopeptide to examine the conformation of the Val residue in **1** [49,58,59]. Specifically, the Alder-ene reaction of cyclohexadiene and the Diels-Alder reaction of cyclopentadiene on N-(Cbz)azoglycinyl-proline benzhydrylamide **5** gave the unsaturated azapeptides **6** and **7**, which were hydrogenated with concomitant removal of the benzyloxycarbonyl group, coupled to N-protected alanine, and deprotected (Scheme 1, [49]). Considering the tolerance of N-methyl-alanine for alanine in the terminal position [60], a similar approach was used to prepare N-methyl analog **4** by hydrogenation of azapeptide **7**, coupling of the resulting aza-cyclohexylglycinyl-L-proline benzhydrylamide (**8**) to N-(Boc)-N-methylalanine using PyBop and cleavage of the Boc group with TFA in DCM. In cultured MCF7 breast cancer cells, aza-cyclohexylglycine analog **3** induced up to 60% cell death relative to vehicle [49].

#### 3.1.1. Effect of Smac Mimic Administration In Vivo

**Figure 2:**
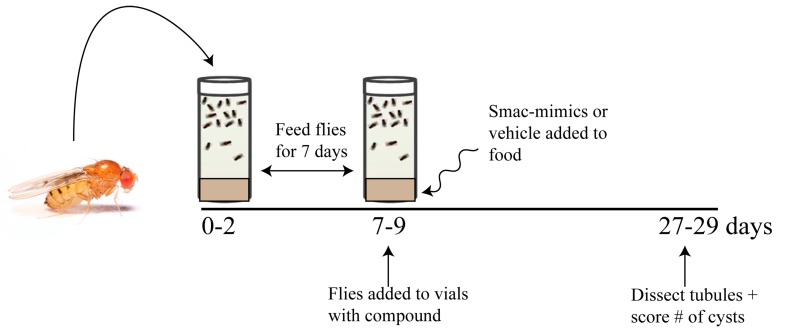
Testing protocol for Smac-mimic efficacy in cyst reduction. *Ore^R^* wild-type, *BicC^Δ/YC33^*, and *BicC^Δ/IIF34^* flies (0–2 days old) were placed in food-containing vials at age 0–2 days and transferred into fresh vials every three days. Once the age of 7–9 days was reached, flies were placed in vials containing one of each of the Smac-mimics or vehicle control, respectively. Cysts were scored on the micro-dissected MTs after 20 days, when flies reached age 27–29 days.

**Figure 3:**
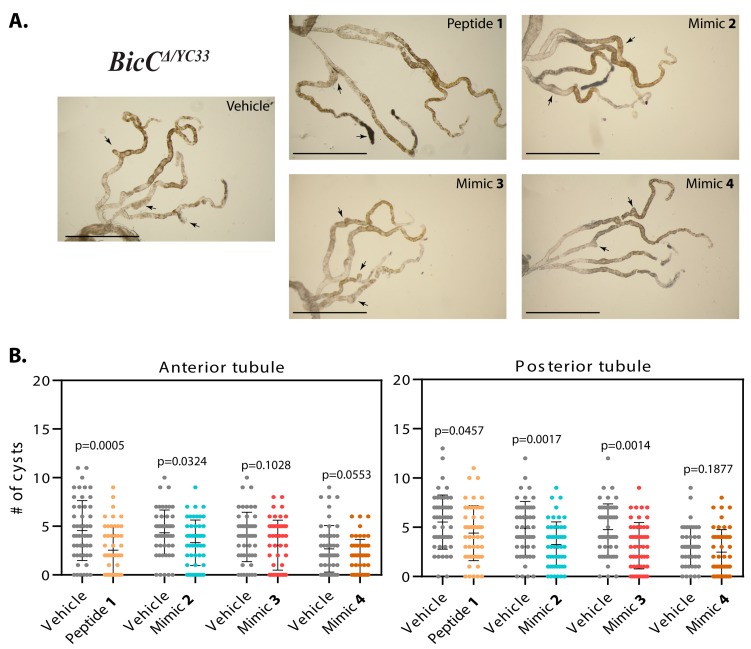
Smac-mimics reduced cysts in *BicC^Δ/YC33^* flies. (**A**). Representative Malpighian tubules micro-dissected from *BicC^Δ/YC33^* flies treated with either vehicle or analogs **1**, **2**, **3**, and **4** (indicated) were photographed *ex vivo*. Arrows indicate cysts. In each image, anterior pairs are at the top, posterior pairs at the bottom. Scale bar: 1 mm. (**B**). Nested plots depicting number of cysts found in the anterior and posterior tubule pairs of vehicle- and Smac mimic-treated cystic flies (indicated), with mean and standard deviation. *P* values (with Welch’s correction) are indicated. Treatments are indicated with color: vehicle, grey; mimic **1**, sepia; **2**, blue; **3**, dark pink; **4**, brown.

**Figure 4:**
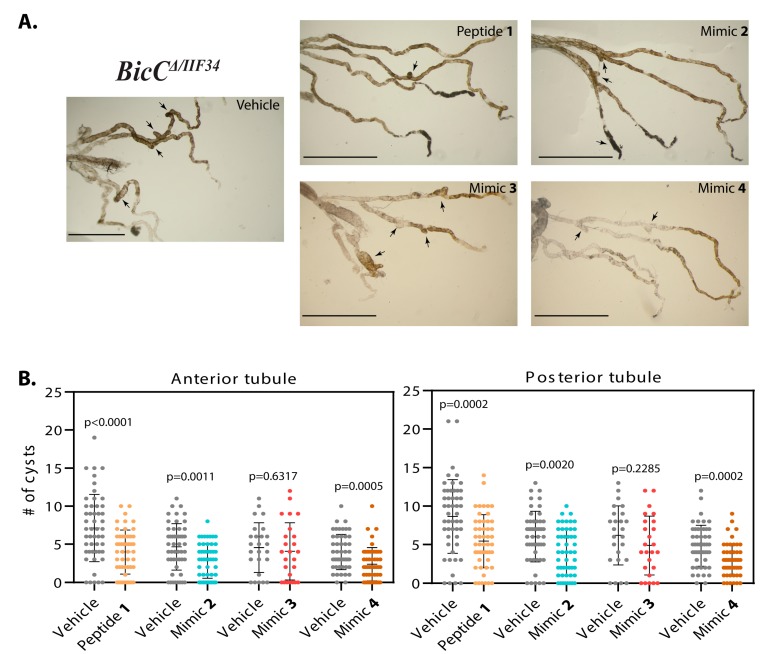
Smac-mimics reduced cysts in *BicC^Δ/IIF34^* flies. (**A**). Representative Malpighian tubules micro-dissected from *BicC^Δ/IIF34^* flies treated with either vehicle or analogs **1**, **2**, **3**, and **4** (indicated) were photographed *ex vivo*. Arrows indicate cysts. In each image, anterior pairs are at the top, posterior pairs at the bottom. Scale bar: 1 mm. (**B**). Nested plots depicting number of cysts found in the anterior and posterior tubule pairs of vehicle- and Smac mimic-treated cystic flies (indicated), with mean and standard deviation. *P* values (with Welch’s correction) are indicated. Treatments are indicated with color: vehicle, grey; mimic **1**, sepia; **2**, blue; **3**, dark pink; **4**, brown.

**Figure 5:**
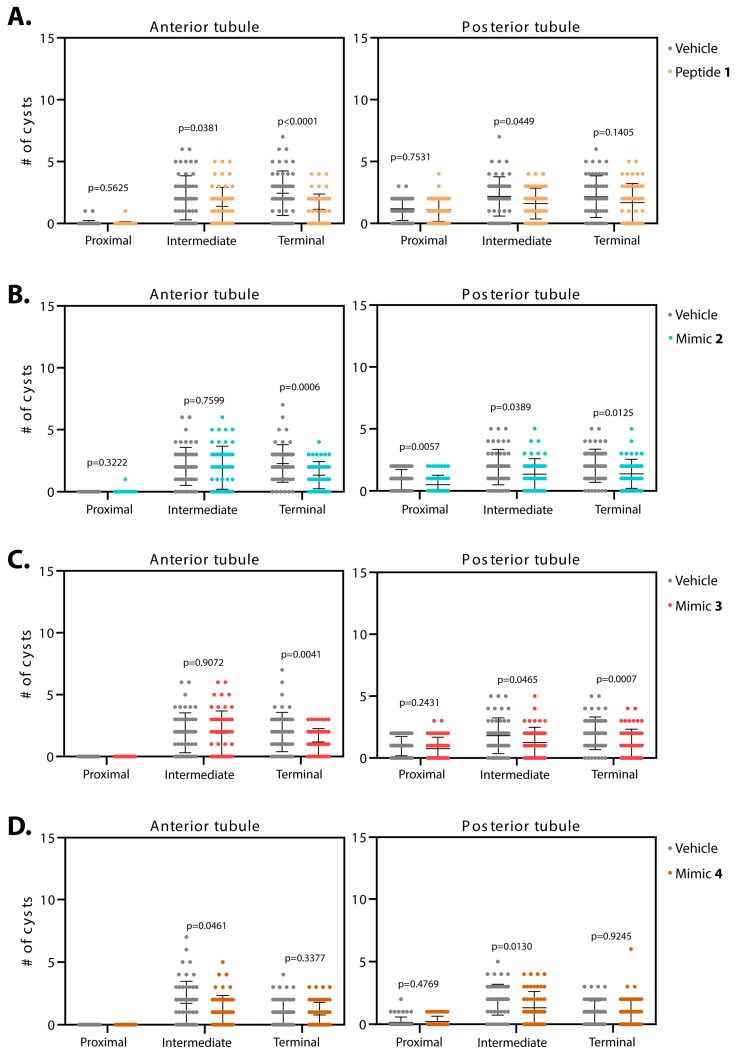
Smac-mimics reduce cysts in *BicC^Δ/YC33^* flies with varying regional specificity. Malpighian tubules from 50 *BicC^Δ/YC33^* flies were micro-dissected after 20 days of treatment (age 27–29 days). Cysts were scored *ex vivo*. For each fly, cysts numbers were recorded for the terminal, intermediate and proximal region of the Malpighian tubules, independently for the anterior (left) and posterior (right) tubules. Flies were administered either vehicle (water, grey) or Smac mimics **1** (**A**, sepia), **2** (**B**, blue), **3** (**C**, dark pink), and **4** (**D**, brown). Mean and standard deviation are indicated. Analogs **1** and **2** best reduced tubular cysts, mimic **3** was effective at reducing cysts in the terminal region of the anterior tubules and mimic **4** had a mild effect in the intermediate region of both tubule pairs. *P* values (with Welch’s correction) are indicated. *BicC^Δ/YC33^* flies exhibit the milder cystic defects.

**Figure 6:**
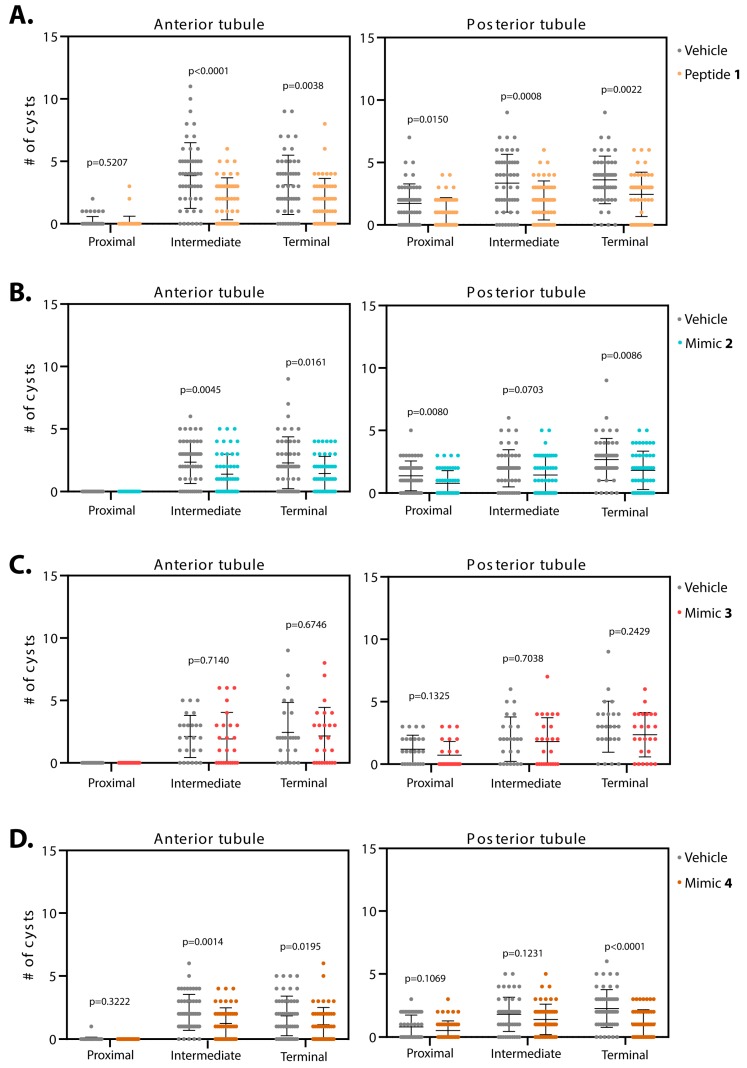
Smac-mimics reduce cysts in *BicC^Δ/IIF34^* flies with varying regional specificity. Malpighian tubules from 25 to 50 *BicC^Δ/IIF34^* flies were micro-dissected after 20 d of treatment (age 27–29 days). Cysts were scored *ex vivo*. For each fly, cysts numbers were recorded for the terminal, intermediate and proximal region of the Malpighian tubules, independently for the anterior (left) and posterior (right) tubules. Flies were administered either vehicle (water, grey) or Smac mimics **1** (**A**, sepia), **2** (**B**, blue), **3** (**C**, dark pink), and **4** (**D**, brown). Mean and standard deviation are indicated. Peptide 1 was the most effective at reducing cysts in the treated tubules. Mimics **2** and **4** showed great improvements in all regions except for the intermediate region of the posterior tubules and mimic **3** showed a very mild to no effect in cyst reduction. *P* values (with Welch’s correction) are indicated. *BicC^Δ/IIF34^* flies exhibit the more severe cystic defects.

We have previously reported that flies mutant in the *BicC* gene recapitulate key features of PKD [3]. Considering that Smac mimics showed efficacy at reducing cysts in a rat PKD model [42], the results of Smac mimic administration were tested in the *BicC* cystic flies using two different allelic combinations. *BicC* mutant flies were obtained from genetic crosses of heterozygote parents consisting of CyO-balanced flies containing *Df(2L)RA5* (Δ, a *BicC*-encompassing deletion), and either the *BicC^YC33^* or *BicC^IIF34^* alleles similarly balanced with CyO. The *BicC^Δ/YC33^* fly exhibits milder cystic defects than the *BicC^Δ^^/IIF34^* fly [3]. Straight-winged mutants (*BicC^Δ/YC33^*, *BicC^Δ/IIF34^*) from crosses were selected within two days of eclosion to yield pools of 0–2 days old flies and aged for seven days (7–9 days old), during which time the flies were kept well-fed by transferring to fresh vials twice (Figure 2). Aged sibling flies were divided into fresh vials containing food spiked with either vehicle (water, 50 µL) or one of the Smac analogs **1**–**4** at 20 μM (50 µL). Flies were transferred into identical fresh vials every three days. After 20 days of treatment, the Malpighian tubules were micro-dissected from 27–29 day old flies and analyzed ex vivo. Per each condition, cystic deformities were scored using 25–50 female *BicC^Δ/IIF34^* and *BicC^Δ/YC33^* flies (i.e., 50–100 anterior and 50–100 posterior tubules). For each fly, cysts which were found in the two anterior and two posterior tubules were scored and charted as nested plots using the Prism 8.0 (Graphpad) software (Figures 3–6). Vehicle-treated flies presented several cysts in both the anterior and posterior MTs, especially in the terminal and intermediate regions, and fewer cysts in the proximal region, as reported previously [3]. Similar to PKD patients [1], a variable number of cysts was found in different individuals [3]. In general, Smac-mimics appeared to reduce tubular cysts in both *BicC* allelic combinations (Table 1, Table 2).

Effects of Smac mimic administration were recorded as overall cyst reduction (Table 1, Table 2). Nested plots were used to represent individual variability in cyst number in the analyzed fly populations. Administration of peptide **1** to *BicC^Δ/YC33^* flies (*n* = 50) reduced cystic deformities respectively by 44% and 20% (228 vs. 127 cysts and 276 vs. 220 cysts, *p* = 0.0005 and 0.0457) in the anterior and posterior tubules, respectively (Table 1, Figure 3A,B). Administration of mimic **2** to *BicC^Δ/YC33^* flies (*n* = 50) reduced tubular cysts by 24% and 34% (216 vs. 165 cysts, *p* = 0.0324 and 244 vs. 162 cysts, *p* = 0.0017) in the anterior and posterior tubules, respectively (Table 1, Figure 3A,B). Administration of mimic **3** to *BicC^Δ/YC33^* flies (*n* = 50) reduced cysts in the anterior and posterior tubules respectively by 21% and 34% (195 vs. 153 cysts, *p* = 0.1028 and 238 vs. 156 cysts, *p* = 0.0014, Table 1, Figure 3A,B). Finally, administration of mimic **4** to *BicC^Δ/YC33^* flies (*n* = 50) reduced cysts in the anterior and posterior tubules respectively by 31% and 18% (134 vs. 93 cysts, *p* = 0.0553 and 152 vs. 124 cysts, *p* = 0.1877, Table 1, Figure 3A,B).

The analogs were next administered to the *BicC^Δ/IIF34^* flies carrying the allelic combination which produced a more severe phenotype. Treatment with peptide **1** decreased cystic deformations respectively by 44% and 37% (total 357 vs. 199 cysts and 433 vs. 272 cysts, *p* < 0.0001 and 0.0002, respectively) in the anterior and posterior tubules of the micro-dissected *BicC^Δ/IIF34^* flies (*n* = 50, Table 2, Figure 4A,B). Administration of mimic **2** to *BicC^Δ/IIF34^* flies (*n* = 50) reduced cystic deformations by 39% in the anterior (233 vs. 142 cysts, *p* = 0.0011) and by 33% in the posterior (302 vs. 202 cysts, *p* = 0.0020) tubules (Table 2, Figure 4A,B). Administration of mimic **3** to *BicC^Δ/IIF34^* flies (*n* = 25) had a milder and highly variable effect on the renal tubules and reduced cystic deformities by 11% (114 vs. 102 cysts, *p* = 0.6317) and 21% (155 vs. 122 cysts, *p* = 0.2285) in the anterior and posterior tubules respectively (Table 2, Figure 4A,B), below statistical relevance thresholds. Finally, administration of mimic **4** to *BicC^Δ/IIF34^* flies (*n* = 50) reduced cysts in the anterior and posterior tubules by 40% (199 vs. 119 cysts, *p* = 0.0005) and 38% (243 vs. 151 cysts, *p* = 0.0002) respectively (Table 2, Figure 4A,B). Parallel respective administration of analogs **1**–**4** to control, non-cystic *Ore^R^* wild type flies did not change MT morphology.

Distinct MT regions in both the anterior and posterior tubules appeared to respond differentially to the Smac mimics. The cyst location was thus specifically mapped in the proximal, intermediate and terminal regions of the anterior and posterior tubules respectively (Table 3 and Table 4), and individual variable cyst numbers similarly plotted (Figure 5 and Figure 6). Absolute numbers for the cysts in each region described below are listed in Table S2.

#### 3.1.2. Smac Mimics Differentially Affect Distinct Regions of the MTs

Administration of peptide **1** to the milder allelic combination *BicC^Δ/YC33^* (*n* = 50) reduced cysts in the terminal and intermediate regions of the anterior tubules respectively by 53% and 34% (total 122 vs. 57, 104 vs. 69 cysts, *p* < 0.0001 and *p* = 0.0381, Table 3, Figure 5A). In the proximal region of the anterior tubules, two cysts were found in the control versus one cyst in the treated samples, precluding statistical analyses. In the posterior tubules, administration of peptide **1** diminished cysts in the intermediate region by 26% (total 109 vs. 80 cysts, *p* = 0.0449). In the terminal and proximal regions, a trend toward decreased cysts was observed, albeit without reaching a statistical threshold for significance (respectively 108 vs. 84 cysts, 22% reduction, *p* = 0.1405, and 59 vs. 56 cysts, 5% reduction, *p* = 0.7531, Table 3, Figure 5A).

Administration of peptide **1** to *BicC^Δ/IIF34^* flies reduced cysts in the anterior tubules by 40% and 48% (total 156 vs. 94 and 193 vs. 100 cysts, *p* = 0.0038 and *p* < 0.0001) in the terminal and intermediate regions, respectively (Table 4, Figure 6A). In the proximal region of the anterior tubules, a trend of cyst reduction was observed, albeit fewer cysts (eight and five in vehicle- and peptide **1**-treated tubules) and high individual variability precluded a margin of confidence. In the posterior tubules, administration of peptide **1** reduced cysts in the terminal, intermediate and proximal regions by 32%, 41% and 39% (total 180 vs. 122, 167 vs. 98, 86 vs. 52 cysts, *p* = 0.0022, 0.0008 and 0.0150), respectively (Table 4, Figure 6A).

Treatment with mimic **2** diminished cysts in the terminal region of the anterior tubules of *BicC^Δ/YC33^* flies (*n* = 50) by 41% (114 vs. 67 cysts, *p* = 0.0006). In the intermediate region, a mild 5% reduction was observed that did not approach a statistical significance threshold (102 vs. 97 cysts, *p* = 0.7599, Table 3, Figure 5B). Precluding statistical analyses, no (control) and one cyst (mimic **2**-treated) were detected in the proximal region of the anterior tubules. In contrast, in the posterior tubule, administration of mimic **2** reduced cysts in the terminal, intermediate and proximal regions by 32%, 29% and 47% (101 vs. 69 cysts, *p* = 0.0125, 96 vs. 68 cysts, *p* = 0.0389 and 47 vs. 25 cysts, *p* = 0.0057), respectively (Table 3, Figure 5B).

Administration of mimic **2** to the more severely cystic *BicC^Δ/IIF34^* flies (*n* = 50) reduced cysts in the terminal and intermediate regions of the anterior tubules by 37% and 41% (average 115 vs. 72 cysts, *p* = 0.0161, 118 vs. 70 cysts, *p* = 0.0045), respectively. No cysts were found in the proximal regions of control and treated tubules (Table 4, Figure 6B). Administration of mimic **2** reduced cysts in the terminal and proximal regions of the posterior tubules by 32% and 43% (134 vs. 91 cysts, *p* = 0.0086 and 69 vs. 39 cysts, *p* = 0.0080), respectively. The intermediate region displayed a 27% reduction that approached statistical significance threshold (99 vs. 72 cysts, *p* = 0.0703, Table 4, Figure 6B).

After treatment with mimic **3**, the *BicC^Δ/YC33^* flies (*n* = 50) harbored 40% fewer cysts in the terminal region of the anterior tubules (99 vs. 59 cysts, *p* = 0.0041). In the intermediate region, the effect was negligible (2% reduction, 96 vs. 94 cysts, *p* = 0.9072). No cysts were detected in the proximal region of both control and treated tubules (Table 3, Figure 5C). On the contrary, administration of mimic **3** reduced strongly cysts in the terminal and intermediate regions of the posterior tubules by 45% and 30% (100 vs. 55 cysts, *p* = 0.0007 and 90 vs. 63 cysts, *p* = 0.0465), respectively. The proximal region displayed a trend towards cyst reduction with high individual variability (21% reduction, 48 vs. 38 cysts, *p* = 0.2431, Table 3, Figure 5C).

Mimic **3** did not ameliorate the more severely cystic *BicC^Δ/IIF34^* flies (*n* = 25). After treatment with mimic **3**, the *BicC^Δ/IIF34^* flies displayed respectively 11% and 9% (61 vs. 54 cysts, *p* = 0.6746 and 53 vs. 48 cysts, *p* = 0.7140) fewer cysts in the terminal and intermediate regions of the anterior tubules; no cysts were observed in the proximal region of both control and treated tubules (Table 4, Figure 6C). Similarly, mimic **3** caused respectively 21%, 10% and 40% (75 vs. 59 cysts, *p* = 0.2429, 50 vs. 45 cysts, *p* = 0.7038 and 30 vs. 18 cysts, *p* = 0.1325) reductions in cysts in the terminal, intermediate and proximal regions of the posterior tubules (Table 4, Figure 6C).

Mimic **4** reduced cysts in the intermediate region of the anterior tubules of *BicC^Δ/YC33^* flies (*n* = 50) by 36% (85 vs. 55 cysts, *p* = 0.0461). In the terminal region, a 20% decrease was observed, that did not reach a threshold of statistical significance (49 vs. 39 cysts, *p* = 0.3377). No cysts were detected in the proximal region of both control and treated tubules (Table 3, Figure 5D). Treatment with mimic **4** reduced cysts in the intermediate region of the posterior tubules by 33% (98 vs. 66 cysts, *p* = 0.0130), but did not reduce cysts in the terminal and proximal regions (Table 3, Figure 5D).

Upon treatment with mimic **4**, cysts were lessened in the terminal and intermediate regions of the anterior tubules of *BicC^Δ/IIF34^* flies (*n* = 50) by 38% and 42% (92 vs. 57 cysts, *p* = 0.0195 and 106 vs. 62 cysts, *p* = 0.0014), respectively (Table 4, Figure 6D). Precluding statistical analysis, only one cyst was detected in the proximal region of control tubules and no cysts in the treated tubules. Administration of mimic **4** reduced cysts in the terminal region of the posterior tubules by 51% (113 vs. 55 cysts, *p* < 0.0001). Less cysts were also scored in the intermediate and proximal regions, although values did not reach the significance threshold (respectively 22% reduction, 90 vs. 70 cysts, *p* = 0.1231, and 35% reduction, 40 vs. 26 cysts, *p* = 0.1069, Table 4, Figure 6D).

Ineffective cyst reduction could be partly due to flies refusing to ingest the Smac mimics. To assess their ingestion, the analogs were mixed with food and dye, and fed to the flies for four days. The green dye could be seen through the semi-transparent abdominal cuticle of the *BicC* flies for all mimics, confirming analog ingestion (Figure S1). As a measure of hydrophilicity, the clogP values were calculated for the different Smac mimics (Figure 1) and found to be sufficiently low to be consistent with absorption: **1** (0.7), **2** (3.9), **3** (5.0), **4** (5.4). Together these results support the conclusion that the Smac analogs may have differential activities and/or processing.

## 4. Discussion

A systematic analysis of the influences of the Smac mimic H-Ala-Val-Pro-Ile-NH_2_ (**1**) and constrained analogs **2**–**4** on renal cystogenesis has been performed using the novel *BicC* fly model to recapitulate features of PKD [3]. Two allelic combinations for *BicC* were used that yield cystic phenotypes of different severity, namely *BicC^Δ/YC33^* and *BicC^Δ/IIF34^*. Previously, the Smac mimic GT13072 reduced cystogenesis in a rat ADPKD model [42]. In the *BicC* flies, Smac mimics **1**–**4** ameliorated similarly the cystic condition with the strongest effects displayed in the more severely affected *BicC^Δ/IIF34^* genotype. Peptide **1** exhibited the highest overall efficacy reducing cyst occurrence by 20–44% across genotypes at the anterior and posterior tubules. Moreover, aza-methanopipecolate **2** caused a 24–39% reduction of cysts. Aza-cyclohexylglycine **3** displayed least efficacy, but still improved significantly the posterior tubules of the *BicC^Δ/YC33^* flies. The related *N*-methyl-alaninyl-aza-cyclohexylglycine analog **4** exhibited differential activity in the two *BicC* mutants, reducing cysts by ~40% in both tubules of the *BicC^Δ/IIF34^* flies. In contrast, analog **4** only showed a trend towards reducing cysts in the anterior tubule of the *BicC^Δ/YC33^* flies close to the significance threshold (*p* = 0.0553). The Smac mimics tested appeared to differentially affect the anterior and posterior tubule pairs overall, consistent with the report of the latter having distinct transcriptomes [10] and thus different physiological specialization. Mimics **1, 2** and **3** were found to induce death of 20% (**1**, **2**) and 60% (**3**) of cultured MCF7 adenocarcinoma cells [49]. Their efficacy in other PKD models and patients is unknown. Smac mimics function in context-dependent ways, likely through different IAPs to affect apoptosis via several mechanisms [12]. Due to its methyl group, *N*-methyl-alaninyl-aza-cyclohexylglycine analog **4** was expected to be more stable than aza-cyclohexylglycine **3** in vivo [61,62,63], which was consistent with the observed higher cyst-reducing activity.

The tubular sections responded differentially to treatment with the Smac mimics. The terminal region consistently exhibited better improvement, especially in the weaker *BicC^Δ/YC33^* allelic combination. The flies were shown to ingest the Smac mimics. The clogP values of mimics **1**–**4** were also within the range consistent with effective absorption. The distinct pharmacological responses observed at different regions of the MTs may be related to variability in absorption, metabolism and response to Smac mimics within cystic cells in such regions. Regional specialization of fly MTs has been observed despite the tubular epithelium being composed by only two major cell types (reviewed in [9,64]). Cystogenesis may perturb cells and reduce the threshold for initiating cell death pathways either through caspase-dependent apoptosis or the TNF signaling pathway. The contribution of apoptosis to the early phases of ADPKD is a matter of debate [65]; however, the TNF pathway has been implicated in ADPKD-type renal cystogenesis and suggested to be the primary target of Smac mimics in a rat model of *Pkd1*-dependent ADPKD [42]. The results presented here predict that the *BicC* mutation may feature dysregulated TNF signaling in the epithelial cells of the MTs, as observed in ADPKD-type cystogenesis. The expression of TNF (Eiger) and TNF pathway components in the MT and their respective contributions to tubular function are however unknown. The human *BicC* orthologue *BICC1* has been found to be genetically downstream of the main *PKD1* gene [3]. The pharmacological response to Smac mimics was herein demonstrated to be conserved in the fly illustrating further the phenotypic and molecular similarities between *PKD1*-induced and *BicC*-induced renal cystogenesis.

Notably, pharmacological binding sites have been found to be conserved in *Drosophila* [66]. Chemical probing in the fly in vivo may thus rapidly pinpoint the involvement of specific pathways with complementarity to genetic analyses, indicate conserved biological activity of drug-prototypes, and provide a rapid read-out for effectiveness of pharmacological modulation of specific pathways involved in cystic pathogenesis. The Smac mimics affected different tubular regions differentially. Considering that such specificity may be conserved to humans, the development of personalized pharmacological treatments for cystic renal diseases such as PKD will benefit from precise knowledge of the cyst-ameliorating potential of different Smac mimics.

## Figures and Tables

**Table 1:** Overall cyst reduction upon Smac-mimic treatment of *BicC^Δ/YC33^* flies.

	Anterior Tubule				Posterior Tubule			
Mimic	Cyst # Vehicle	Cyst # Treated	% Reduction	*p* Value	Cyst # Vehicle	Cyst # Treated	% Reduction	*p* Value
**1**	228	127	44%	0.0005	276	220	20%	0.0457
**2**	216	165	24%	0.0324	244	162	34%	0.0017
**3**	195	153	*21%*	*0.1028*	238	156	34%	0.0014
**4**	134	93	*31%*	*0.0553*	152	124	*18%*	*0.1877*

*P* values > 0.05 and corresponding percentages are italicized.

**Table 2:** Overall cyst reduction upon Smac-mimic treatment of *BicC^Δ/IIF34^* flies.

	Anterior Tubule				Posterior Tubule			
Mimic	Cyst # Vehicle	Cyst # Treated	% Reduction	*p* Value	Cyst # Vehicle	Cyst # Treated	% Reduction	*p* Value
**1**	357	199	44%	<0.0001	433	272	37%	0.0002
**2**	233	142	39%	0.0011	302	202	33%	0.0020
**3**	114	102	*11%*	*0.6317*	155	122	*21%*	*0.2285*
**4**	199	119	40%	0.0005	243	151	38%	0.0002

*P* values > 0.05 and corresponding percentages are italicized.

**Table 3:** Percentage of cyst reduction upon treatment of *BicC^Δ/YC33^* flies.

Smac-Mimic	Anterior Tubule			Posterior Tubule		
	Prox.	Int.	Term.	Prox.	Int.	Term.
**1** (*n* = 50)	n/s	34% *p* = 0.0381	53% *p* < 0.0001	*5%* *p* = *0.7531*	26% *p* = 0.0449	*22%* *p* = *0.1405*
**2** (*n* = 50)	n/s	*5%* *p = 0.7599*	41% *p* = 0.0006	47% *p* = 0.0057	29% *p* = 0.0389	32% *p* = 0.0125
**3** (*n* = 50)	n/a	*2%* *p = 0.9072*	40% *p* = 0.0041	*21%* *p* = *0.2431*	30% *p* = 0.0465	45% *p* = 0.0007
**4** (*n* = 50)	n/a	36% *p* = 0.0461	*20%* *p* = *0.3377*	n/s *p* = *0.4769*	33% *p* = 0.0130	n/s *p* = *0.9245*

*P* values > 0.05 and corresponding percentages are italicized; n/s = not significant; n/a = no cysts.

**Table 4:** Percentage of cyst reduction upon treatment of *BicC^Δ/IIF34^* flies.

Smac-Mimic	Anterior Tubule			Posterior Tubule		
	Prox.	Int.	Term.	Prox.	Int.	Term.
**1** (*n* = 50)	n/s	48% *p* < 0.0001	40% *p* = 0.0038	39% *p* = 0.0150	41% *p* = 0.0008	32% *p* = 0.0022
**2** (*n* = 50)	n/a	41% *p* = 0.0045	37% *p* = 0.0161	43% *p* = 0.0080	*27%* *p* = *0.0703*	32% *p* = 0.0086
**3** (*n* = 25)	n/a	*9%* *p* = *0.7140*	*11%* *p* = *0.6746*	*40%* *p* = *0.1325*	*10%* *p* = *0.7038*	*21%* *p* = *0.2429*
**4** (n = 50)	n/s	42% *p* = 0.0014	38% *p* = 0.0195	*35%* *p* = *0.1069*	*22%* *p* = *0.1231*	51% *p* < 0.0001

*P* values > 0.05 and corresponding percentages are italicized; n/s = not significant; n/a = no cysts.

## Supplementary Materials

The following are available online at https://www.mdpi.com/2227-9059/7/4/82/s1. Supplementary file S1: spectral and chromatographic characterization of mimic 4, Table S1: Cystic index analysis raw data, Table S2: Regional cystic index for BicC flies. Figure S1: Analog feeding control.
