# Supplementary material for: Cyst Reduction in a Polycystic Kidney Disease Drosophila Model Using Smac Mimics
Source: Biomedicines. 2019 Oct 18;7(4):82. doi: 10.3390/biomedicines7040082 (PMC6966561; doi:10.3390/biomedicines7040082)
Supplement: Supplementary file 1 [file biomedicines-07-00082-s001.zip › supl for XML/biomedicines-610868-supl-proofed.pdf]

EXPERIMENTAL SECTION

General Methods

Chemicals were used as received from commercial sources without further purification unless stated otherwise. All glassware was stored in the oven or flame-dried and let cool under an inert atmosphere prior to use. Anhydrous solvents (DCM, and DMF) were obtained by passage through solvent filtration systems (Glass-Contour, Irvine, CA). Visualization of the developed chromatogram was performed by UV absorbance or staining with ceric ammonium molybdate or potassium permanganate solutions. Silica gel chromatography was performed using 230–400 mesh silica gel (Silicycle), and TLC was on glass-backed silica plates. Nuclear magnetic resonance spectra (^{1}H, ^{13}C) were recorded on Bruker AV 500 spectrometer. ^{1}H NMR spectra were referenced to CD_3OD (3.31 ppm) and ^{13}C NMR spectra were measured in CD_3OD (49.00 ppm) as specified below. Coupling constant, J values were measured in Hertz (Hz) and chemical shift values in parts per million (ppm). Infrared spectra were recorded in the neat on a Perkin Elmer Spectrum One FTIR instrument and are reported in reciprocal centimetres (cm^{-1}). Accurate mass measurements were performed on a liquid chromatography−mass spectrometry (LC−MS) instrument from Agilent Technologies 1200 series in positive electrospray ionization (ESI)-time-of-flight (TOF) mode at the Université de Montréal Mass Spectrometry facility. Sodium and proton adducts ($[M + Na]^+$ and $[M + H]^+$) were used for empirical formula confirmation.

Reagents Used. Aza-Cyclohexaneglycinyl-L-Proline Benzhydrylamide was synthesized according to the literature method (*Biopolymers*, **2016**, *106*, 235-44). *N*-Boc-*N*-methyl-L-alanine and diisopropyl ethyl amine (DIEA) were purchased from Aldrich or Alfa Aesar and used without further purification. Benzotriazol-1-yl-oxytripyrrolidinophosphoniumhexafluorophosphate (PyBop) was purchased from GL Biochem™, recrystallized prior to use from dry CH_2Cl_2/Et_2O (melting point, 156 °C) and stored in the dark.

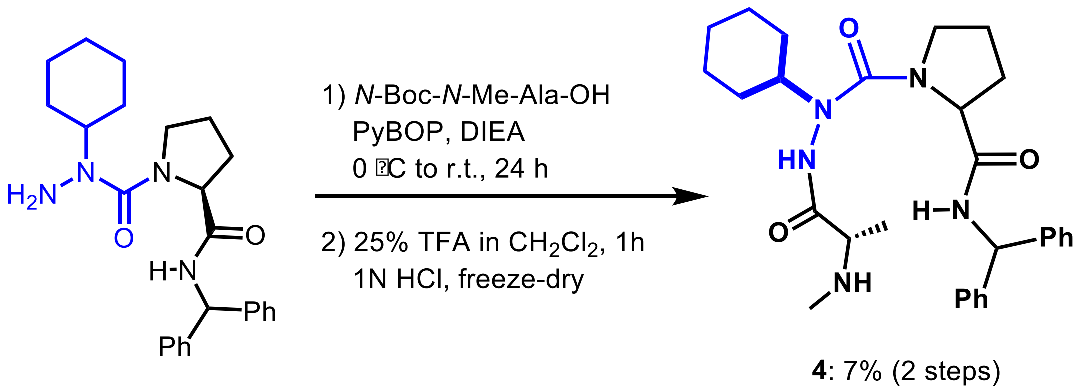

4: 7% (2 steps)

N-methyl Alaninyl-aza-Cyclohexaneglycinyl-L-Proline Benzhydrylamide (**4**). A solution of Aza-Cyclohexaneglycinyl-L-Proline Benzhydrylamide (1 eq., 65 mg, 0.155 mmol) and DIEA (2 eq., 40 mg, 53 μL, 0.309 mmol) was added to a solution of *N*-(tert-butoxycarbonyl)-*N*-methyl-L-alanine (1.2 eq., 38 mg, 0.186 mmol) and PyBOP (1.5 eq., 121 mg, 0.233 mmol) in DMF (3 mL), and stirred overnight. The volatiles were removed under vacuum. The residue was dissolved in EtOAc (10 mL), washed with 5 mL of saturated aqueous $NaHCO_3$, followed by brine (10 mL), dried over Na_2SO_4, filtered, and evaporated. Without further purification, the residue was dissolved in a 25% solution of TFA in dichloromethane (2 mL), and stirred for 2 h. The volatiles were removed under vacuum. The residue was dissolved in CH_2Cl_2 and the solution was evaporated. The residue was suspended in 2 mL of 1N HCl, stirred for 30 min, and freeze-dried to give the hydrochloride salt as off white solid. The crude sample was purified by RP-HPLC on a reverse-phase Gemini® C_{18} column (Phenomenex® Inc., pore size: 110 Å, particle size: 5 μm, 250 x 21.2 mm) using a binary solvent system consisting of a gradient of 5%-60% MeOH (0.1% FA) in water (0.1% FA), with a flow rate of 10.0 mL/min, and UV detection at 214 nm. The desired fractions were combined and freeze-dried to white fluffy powder: azapeptide **2** (5.2 mg, 0.01 mmol, 7%, 2-steps): mp 93-94 °C; ^{1}H NMR (500 MHz, MeOD) δ 8.40 (s, 1H), 7.45 – 7.20 (m, 10H), 6.24 (s, 1H), 4.56 – 4.42 (m, 1H), 4.05 – 3.88 (m, 1H), 3.69 – 3.42 (m, 2H), 2.59 – 2.43 (m, 3H), 2.43 – 2.31 (m, 1H), 2.26 – 2.14 (m, 1H), 2.14 – 2.00 (m, 1H), 2.00 – 1.91 (m, 1H), 1.91 – 1.62 (m, 6H), 1.53 – 1.36 (m, 5H), 1.35 – 1.03 (m, 5H); ^{13}C NMR (125 MHz, MeOD) δ 174.1, 171.2, 161.4, 143.2, 139.1, 129.5 (2C), 129.4 (2C), 129.3 (2C), 128.8 (2C), 128.4, 128.2, 63.6, 57.9, 57.4, 49.6, 31.5, 31.3, 30.8, 26.9, 26.72 (2C), 26.69 (2C), 26.5, 17.5; IR (neat) v_{max}/cm^{-1} 2929, 1638, 1532, 1495, 1449, 1406, 1344, 1323, 1095; HRMS m/z calculated for $C_{29}H_{40}N_5O_3$ $[M+H]^+$ 506.3126; found 506.3139.

^{1}H-NMR, 500 MHz, MeOD

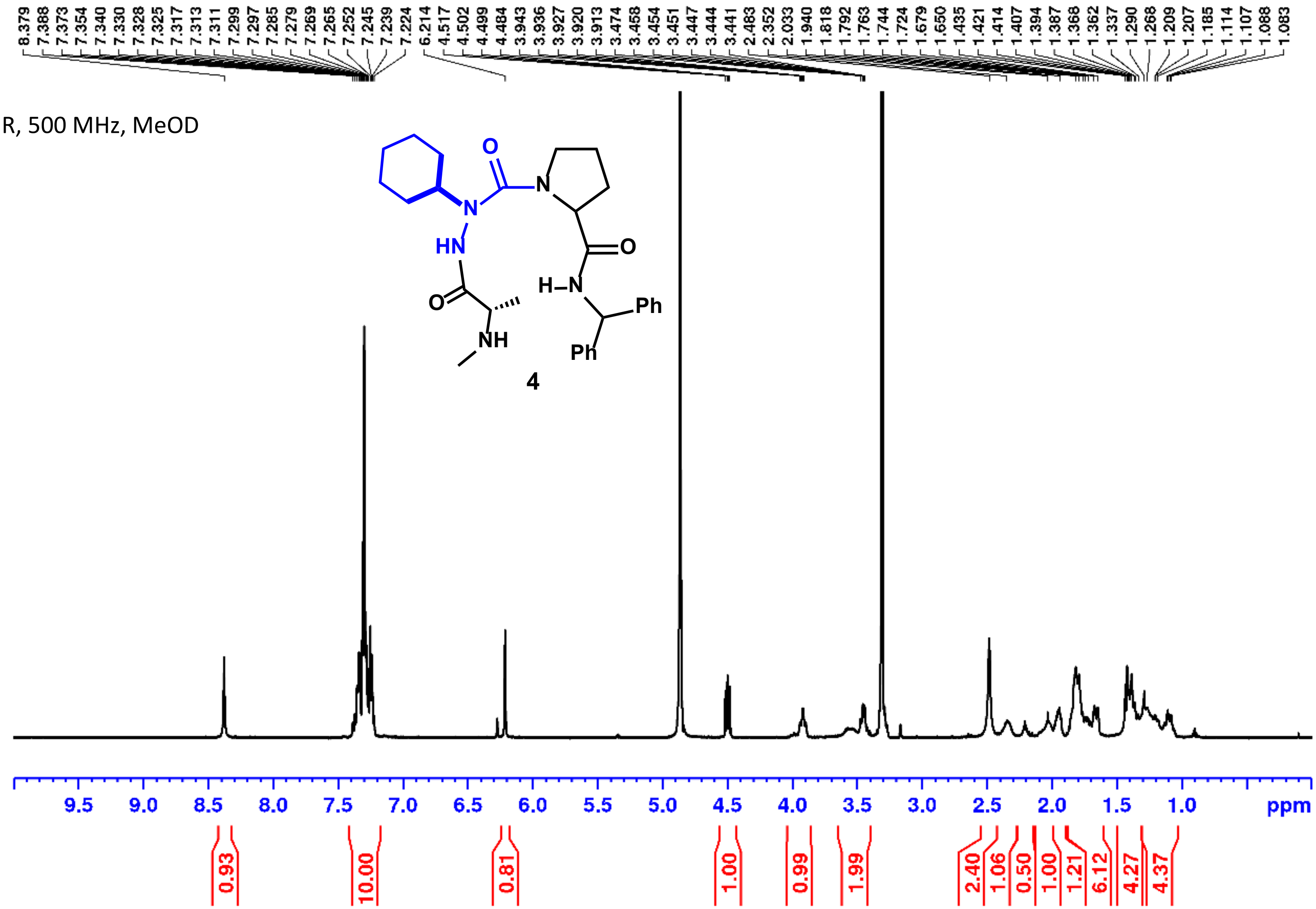

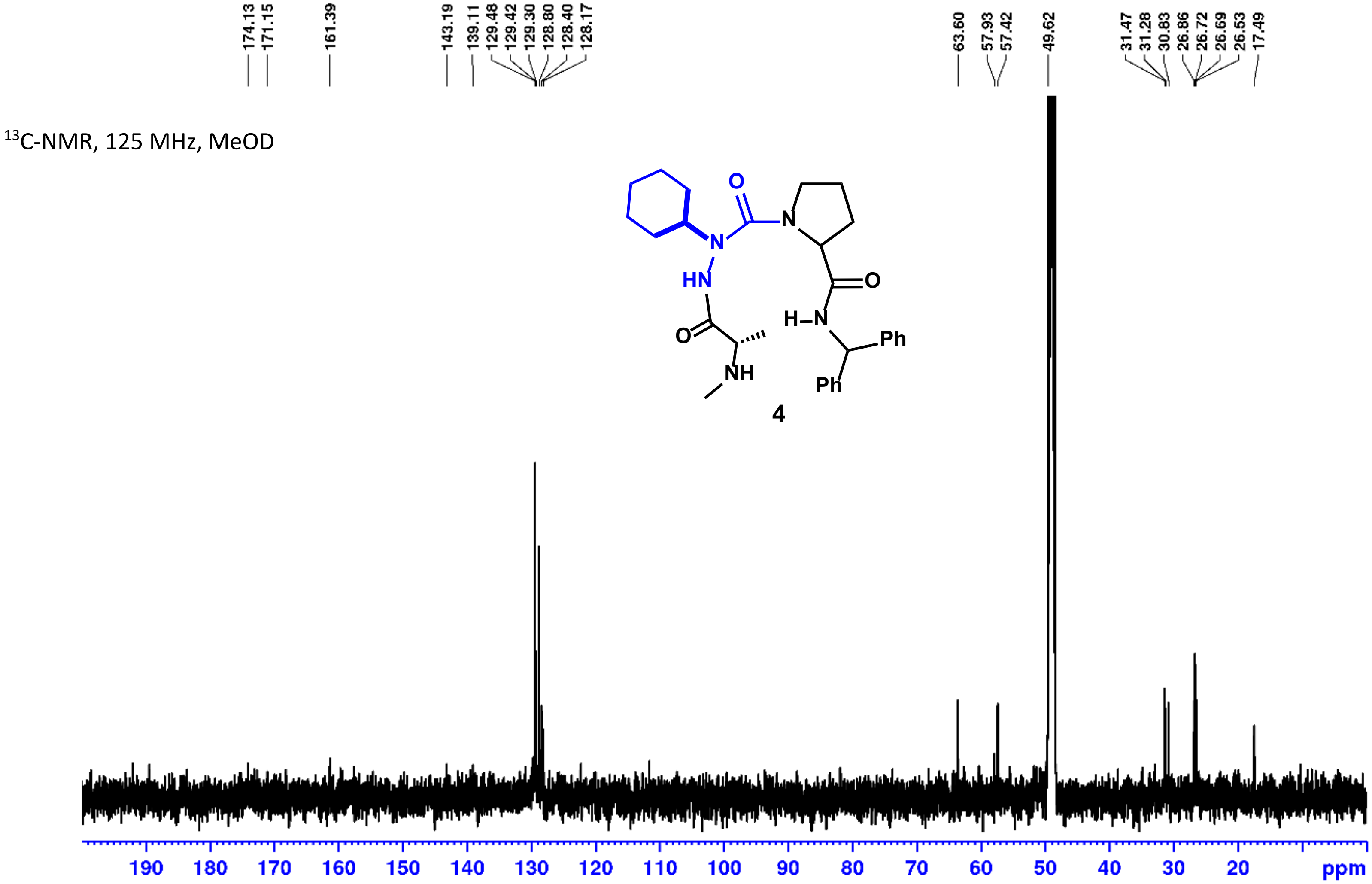
^{13}C-NMR, 125 MHz, MeOD
174.13
171.15
161.39
143.19
139.11
129.48
129.42
129.30
128.80
128.40
128.17
63.60
57.93
57.42
49.62
31.47
31.28
30.83
26.86
26.72
26.69
26.53
17.49
O
N
N
HN
O
H–N
O
NH
Ph
Ph
4
190 180 170 160 150 140 130 120 110 100 90 80 70 60 50 40 30 20 ppm

Rapport de masse exacte

**Data File**	WDL-Rc-N-me-chingle 05.d	**Sample Name**	WDL-Rc-N-me-chingle 05
**Sample Type**	Sample	**Position**	P1-B2
**Analysis Date**	4/9/2018 9:34:03 AM	**User Name**	KG
**Acq Method**	ESI_POS_DI.m	**InstrumentName**	TOF 6224
**Comment**			

MS Spectrum

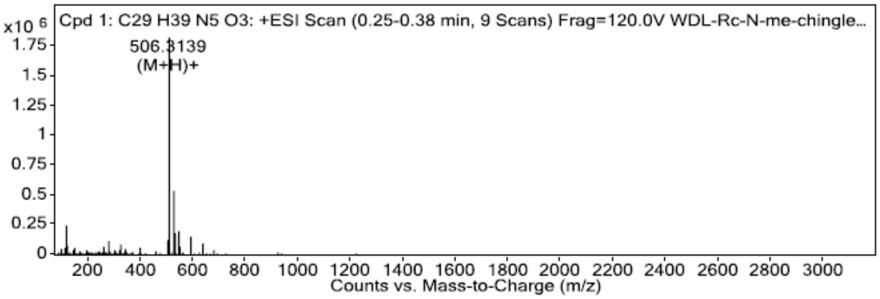

MS Zoomed Spectrum

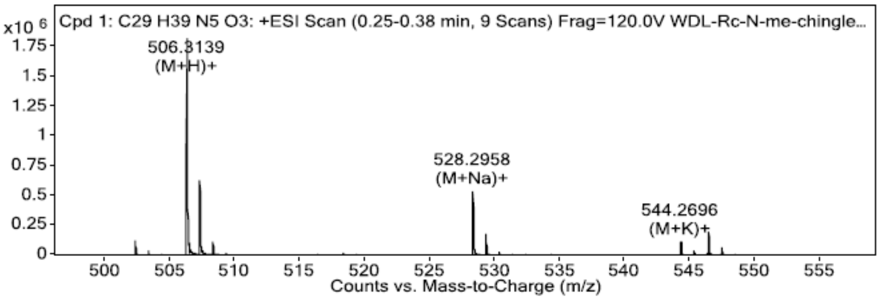

MS Spectrum Peak List

Ion	Formula	Abund	*Expe. m/z*	*Calc. m/z*	Diff(ppm)
(M+H)+	C29H39N5O3	1839043.44	506.3139	506.3126	-2.57
(M+Na)+	C29H39N5O3	545573	528.2958	528.2945	-2.39

Table S2. Scored cyst numbers for each region of the Malpighian tubules as indicated.

Regional cystic index for *BicC*$^{\Delta/YC33}$ flies

	Anterior			Posterior		
	**Proximal**	**Intermediate**	**Terminal**	**Proximal**	**Intermediate**	**Terminal**
Vehicle **1**	2	104	122	59	109	108
Peptide **1**	1	69	57	56	80	84
Vehicle **2**	0	102	114	47	96	101
Mimic **2**	1	97	67	25	68	69
Vehicle **3**	0	96	99	48	90	100
Mimic **3**	0	94	59	38	63	55
Vehicle **4**	0	85	49	8	98	46
Mimic **4**	0	54	39	11	66	47

Regional cystic index for *BicC*$^{\Delta/IIF34}$ flies

	Anterior			Posterior		
	**Proximal**	**Intermediate**	**Terminal**	**Proximal**	**Intermediate**	**Terminal**
Vehicle **1**	8	193	156	86	167	180
Peptide **1**	5	100	94	52	98	122
Vehicle **2**	0	118	115	69	99	134
Mimic **2**	0	70	72	39	72	91
Vehicle **3**	0	53	61	30	50	75
Mimic **3**	0	48	54	18	45	59
Vehicle **4**	1	106	92	40	90	113
Mimic **4**	0	62	57	26	70	55

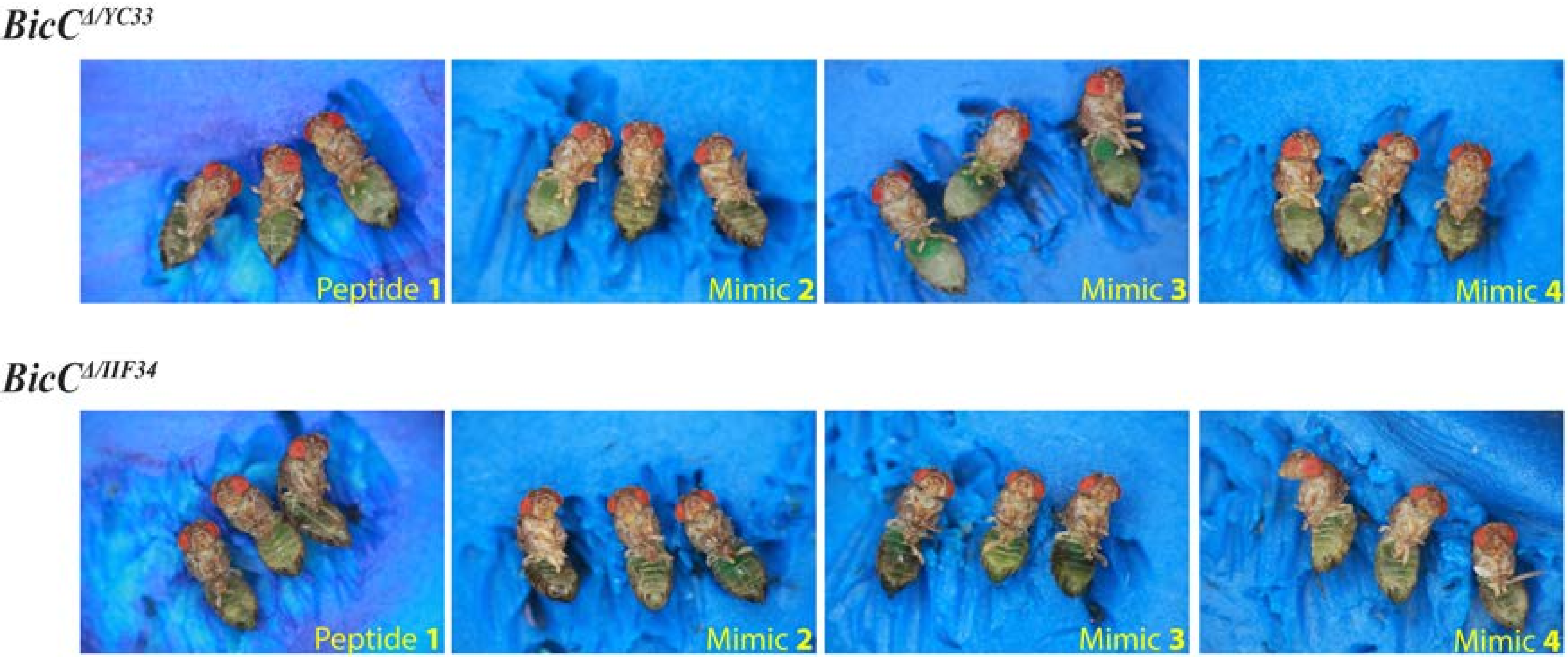

Figure S1. Analog feeding control. *BicC* flies fed Smac mimics mixed with green dye for four days, displayed ingested food contents visible through their semi-transparent abdominal cuticle. Legs were clipped for better visualization.
